# Association of dietary fiber intake with all-cause and cardiovascular mortality in U.S. adults with metabolic syndrome: NHANES 1999–2018

**DOI:** 10.3389/fnut.2025.1659000

**Published:** 2025-09-11

**Authors:** Yafei Guo, Meiling Li, Yueqin Huang

**Affiliations:** Department of Hematology, The Second Affiliated Hospital of Fujian Medical University, Quanzhou, China

**Keywords:** dietary fiber, metabolic syndrome, mortality, cardiovascular disease, NHANES, cohort study

## Abstract

**Background:**

Low dietary fiber intake is common in the US, despite its health benefits. Individuals with metabolic syndrome (MetS), at high cardiovascular risk, may benefit significantly from higher fiber, but its link to mortality in this group is unclear.

**Methods:**

We analyzed prospective data from 10,962 U.S. adults with MetS (NHANES 1999–2018, mean age 58.1). Baseline fiber intake (g/day) was assessed via 24-h recalls. MetS was defined by ATP III criteria. Mortality (all-cause, CVD-specific) was tracked via the National Death Index (median follow-up 102 months). Cox models estimated hazard ratios (HRs) for mortality associated with fiber intake, adjusted for demographics, socioeconomic status, lifestyle, and comorbidities.

**Results:**

Over follow-up, 2,617 deaths occurred (887 CVD-specific). Higher fiber intake was associated with significantly lower mortality. Our analysis suggested a potential threshold effect near 21.7 g/day of fiber intake. Below this, each additional 5 g fiber reduced all-cause mortality risk by 7% (HR = 0.93, 95% CI: 0.91–0.96, *p* < 0.0001). Comparing highest to lowest tertile intake, adjusted HRs were 0.80 (95% CI 0.72–0.89, *p* < 0.0001) for all-cause and 0.61 (0.51–0.73, p < 0.0001) for CVD mortality. Results were robust in sensitivity analyses.

**Conclusion:**

In U.S. adults with MetS, higher dietary fiber intake was associated with significantly lower all-cause and CVD mortality. Benefits were most pronounced at lower intakes, plateauing around 22 g/day, suggesting achieving moderate fiber intake near recommendations offers substantial survival benefits in this high-risk group.

## 1 Introduction

Metabolic syndrome (MetS) is a common metabolic disorder characterized by a cluster of risk factors (abdominal obesity, hypertension, dyslipidemia, and hyperglycemia) that together increase the risk of cardiovascular disease (CVD) and diabetes (1, 2). It is estimated that roughly one-third of U.S. adults meet the criteria for metabolic syndrome (3). Moreover, having MetS significantly elevates the risk of mortality and cardiovascular events. For example, adults with metabolic syndrome have about a 24% higher risk of all-cause death and a 44% higher risk of heart disease mortality compared to those without MetS (4). Given the high prevalence and clinical significance of metabolic syndrome, identifying modifiable lifestyle factors that could improve outcomes in this population is of great importance.

Dietary fiber intake is one such factor that may influence health and longevity in individuals with MetS. Fiber consumption has well-established benefits for cardiovascular and metabolic health, including improvements in blood lipid profiles, blood pressure, glycemic control, and weight management (5, 6). High-fiber diets are associated with lower risk of coronary heart disease, stroke, type 2 diabetes, and certain cancers conditions closely linked to metabolic syndrome (7–9). Nutritional guidelines consistently recommend a high fiber intake (e.g., ~25–38 g per day for adults, varying by age and sex) as part of a healthy diet (10). However, most people in the United States consume far below these recommendations: national data show that ~95% of Americans do not meet the recommended fiber intake, with mean intake only about 15–16 g/day (11). This “fiber gap” has led public health authorities to designate fiber as a shortfall nutrient of concern (12). Improving fiber intake is therefore a priority, especially for high-risk groups like those with metabolic syndrome.

Although previous studies have established a negative correlation between dietary fiber intake and mortality in the general population (13), the specific impact of fiber on mortality outcomes in individuals with metabolic syndrome remains underexplored. Given the high prevalence of metabolic syndrome and its associated health risks (2), understanding the potential benefits of dietary fiber in this high-risk group is crucial for developing targeted public health interventions. Our study aims to fill this gap by providing detailed insights into the relationship between dietary fiber intake and mortality among adults with metabolic syndrome.

Higher dietary fiber has been linked to reduced mortality in general population cohorts. For instance, in the large NIH-AARP study of older adults, those in the highest quintile of fiber intake had a ~ 22% lower risk of total death compared to those in the lowest quintile (14). A 2019 systematic review and meta-analysis of prospective studies similarly found that diets high in fiber were associated with 15–30% lower all-cause and cardiovascular mortality (15). Notably, the greatest risk reductions were observed with fiber intakes in the range of about 25–30 g/day, suggesting a possible threshold effect. Despite this evidence in broader populations, data specifically examining fiber’s impact on mortality among individuals with metabolic syndrome are limited. People with MetS tend to have multiple diet-related risk factors, so they might particularly benefit from fiber, but this needs clarification.

In this context, we aimed to investigate the association between dietary fiber intake and mortality outcomes in a U.S. adults with metabolic syndrome. Using data from NHANES 1999–2018 with up to ~18 years of follow-up, we examined whether higher fiber consumption is linked to lower all-cause mortality (primary outcome) and CVD-specific mortality (secondary outcome) in this high-risk group. We additionally explored whether the relationship is linear or if diminishing returns emerge at higher fiber intakes. We hypothesized that greater fiber intake would be associated with reduced mortality risk in MetS, potentially with a plateau at higher intake levels, consistent with prior research in general populations.

## 2 Methods

### 2.1 Study population

This analysis utilized data from the National Health and Nutrition Examination Survey (NHANES) 1999–2018, a continuous series of cross-sectional surveys employing a complex multistage probability sampling design to obtain a nationally representative of the U.S. civilian, non-institutionalized population. The survey combines detailed health examinations, laboratory tests, and questionnaires, with rigorous quality control measures and standardized protocols to ensure data validity. We pooled ten NHANES cycles (1999–2000 through 2017–2018). The NHANES study was approved by the National Center for Health Statistics (NCHS) Research Ethics Review Board, and all participants provided written informed consent. More details are available at the NHANES website: https://www.cdc.gov/nchs/nhanes/index.html.

**Figure 1:**
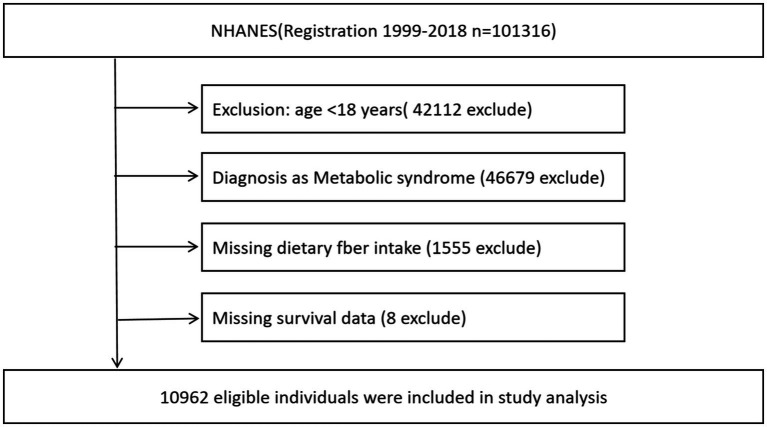
Patient enrollment and exclusion flowchart.

Participants were eligible if they were adults (age ≥18 years) and met the criteria for metabolic syndrome at baseline. Metabolic syndrome was defined according to the National Cholesterol Education Program Adult Treatment Panel III (ATP III) definition (4), requiring at least three of the following: ① elevated waist circumference (≥102 cm in men or ≥88 cm in women), ② elevated blood pressure (≥130/85 mmHg or taking antihypertensive medication), ③ low HDL cholesterol (<40 mg/dL in men or <50 mg/dL in women), ④ elevated fasting triglycerides (≥150 mg/dL or on lipid-lowering therapy), and ⑤ elevated fasting glucose (≥100 mg/dL or on medication for hyperglycemia). We identified 101,316 participants with MetS at baseline across the survey cycles. We further excluded individuals with missing dietary data or implausible energy intake, and those with missing mortality information. The final analytic sample comprised 10,962 adults with metabolic syndrome (Figure 1).

### 2.2 Dietary fiber assessment

Dietary intake was measured in NHANES via 24-h dietary recall interviews administered by trained personnel using the USDA’s Automated Multiple-Pass Method. In most survey cycles, two non-consecutive 24 h recalls were collected (the first in-person and the second by telephone), and nutrient intakes were averaged across the 2 days; in early cycles with one recall, that single day was used. The fibre variable used in this study represents total dietary fibre (grams per day) from all foods and beverages consumed, as calculated by the USDA Food and Nutrient Database for Dietary Studies for each recall day. It includes naturally occurring fibre in foods as well as fibre added during food processing, and does not distinguish between these sources. For analysis, fiber intake was treated both as a continuous variable and as a categorical variable in tertiles. The distribution of fiber intake in this MetS population was right-skewed (mean 15.7 g/day, standard deviation 8.5; median ~14 g/day), with roughly two-thirds of participants consuming <20 g/day.

### 2.3 Mortality outcomes

Mortality status and cause of death were determined by linkage of NHANES participants to the National Death Index (NDI) records through December 31, 2018. Person-time was calculated from the NHANES examination date until death or end of follow-up (Dec 31, 2018), whichever came first. The primary outcome for this study was all-cause mortality (death from any cause). The secondary outcome was cardiovascular disease (CVD) mortality, defined as deaths related to heart disease (ICD-10 codes I00-I09, I11, I13, I20-I51, I60-69) (16). Cause-of-death classification was based on ICD-10 codes provided by the NDI, without independent adjudication. Death certificate data from the NDI were used to assign the cause of death.

### 2.4 Covariates

We included a range of covariates known or suspected to confound the relationship between diet and mortality. Demographic covariates were age (years), sex (male or female), race/ethnicity (categorized as Non-Hispanic White, Non-Hispanic Black, Hispanic, or other/mixed), education level (less than high school, high school/GED, or more than high school), and poverty-income ratio [categorized as poor (<1.0), nearly poor (1.0–1.9), middle income (2.0–3.9), high income (≥4.0), and unknown (not acquired)]. Health behavior covariates included smoking status (never, former, or current smoker), alcohol use (non-drinker, moderate drinker, or heavy drinker; with heavy drinking generally defined as >2 drinks/day for men or >1 drink/day for women) and physical activity [assessed using the Global Physical Activity Questionnaire (GPAQ) and categorized based on meeting (≥600 MET-minutes/week) or not meeting (<600 MET-minutes/week) the physical activity guidelines for adults (17)]. Dietary covariates included total energy intake (kcal) (defined as the average daily caloric consumption from all foods and beverages based on the first 24-h dietary recall interview), total flavanones intake (mg) (defined as the sum of hesperetin, naringenin, and eriodictyol from dietary sources, primarily citrus fruits like oranges and grapefruits), and total flavones intake (mg) (defined as the sum of apigenin and luteolin from dietary sources, commonly found in vegetables such as parsley and celery). Clinical covariates included baseline hypertension status (yes/no, defined by blood pressure ≥130/85 mmHg or antihypertensive medication use, which overlaps with the MetS definition component) and diabetes status (yes/no, defined by self-reported physician diagnosis of diabetes or use of glucose-lowering medication, or fasting plasma glucose ≥126 mg/dL). Because all participants had metabolic syndrome, many had hypertension and/or impaired glucose regulation; however, we retained these as covariates to account for varying severity of metabolic abnormalities. We did not adjust for body mass index (BMI) since central obesity is a defining component of MetS and adjusting for it could constitute adjusting for central obesity, which is a diagnostic criterion for MetS, may introduce collider bias. All covariates were assessed at baseline through NHANES examination and questionnaires.

### 2.5 Statistical analysis

We first described baseline characteristics of the study population, using means (± standard deviation) or medians (interquartile range) for continuous variables and percentages for categorical variables. We used Cox proportional hazards regression to evaluate the association between dietary fiber intake and time to mortality. For our primary models, fiber intake was modeled as a continuous exposure per 5 g/day increment. Non-linearity in the fibre–mortality relationship was assessed using generalized additive models (GAM) and restricted cubic splines. These analyses suggested a potential flattening of the risk reduction at higher fibre intakes. To further explore this, we employed a two-piecewise Cox proportional hazards model with a single change-point (threshold) for fibre’s effect. The threshold was identified in a data-driven manner using a recursive method that tested all possible cut points and selected the value that yielded the highest model likelihood. This approach allowed us to more precisely capture potential non-linear dose–response patterns and to quantify the association separately below and above the inflection point. We performed likelihood ratio tests to compare the fit of the two-piecewise model against a single-line model, as described in previous analyses (18). We report hazard ratios (HRs) with 95% confidence intervals for each segment. We also conducted a categorical analysis dividing participants into tertiles of fiber intake (low, medium, high intake) and comparing mortality risks across these groups using the lowest tertile as the reference. Trend across tertiles was evaluated by modeling the median fiber value in each tertile as a continuous variable.

All models were adjusted for the covariates listed above (age, sex, ethnicity, education, poverty-income ratio, smoking, alcohol, hypertension, diabetes). Adjusted HRs were estimated along with *p*-values (two-tailed), and a significance level of *α* = 0.05 was used. Proportional hazards assumptions were evaluated using Schoenfeld residuals for each covariate (individual tests) and for the model as a whole (global test). For the exposure of interest (dietary fiber intake), the individual test was non-significant (*p* = 0.2039), indicating no evidence of violation. Most covariates also satisfied the assumption; however, hypertension showed a statistically significant result (*p* = 0.0295). The global test was marginally significant (*p* = 0.0295), likely influenced by this variable. Sensitivity analyses excluding hypertension from the model produced results consistent with the main findings. We performed several sensitivity analyses to assess the robustness of our findings. As a sensitivity analysis, we further adjusted the fully adjusted models for total flavanones intake (mg) and total flavones intake (mg) to account for potential confounding by specific phytochemicals associated with fruit and vegetable consumption. The two-sided alpha level was set at 0.05. All the statistical analyses were performed using the EmpowerStats (www.empowerstats.com, X&Y solutions, Inc. Boston MA) and R software version 3.6.1.^1^

## 3 Results

### 3.1 Participant characteristics

A total of 10,962 NHANES participants with metabolic syndrome were included, representing an estimated tens of millions of U.S. adults with MetS over 1999–2018. The mean age was 58.1 years (SD 15.6), and 48% were female. The cohort was ethnically diverse (approximately 63% Non-Hispanic White, 18% Hispanic, 14% Non-Hispanic Black, and 5% other race/ethnicity) (Table 1; Supplementary Table S1).

**Table 1:** Characteristics of study participants by dietary fiber intake tertiles.

Characteristics	Total	Dietary fiber intake, g/day			*p*-value
		Tertile1 0.00–11.29	Tertile 2 11.30–17.49	Tertile 3 17.50–80.00	
Number	10,962	3,650	3,636	3,676	
Age (years)	58.06 ± 15.58	57.60 ± 16.07	58.92 ± 15.74	57.67 ± 14.89	<0.001
Body Mass Index (kg/m^2^)	33.11 ± 6.77	33.25 ± 7.06	33.17 ± 6.62	32.90 ± 6.61	0.062
Poverty income ratio	2.42 ± 1.56	2.11 ± 1.47	2.45 ± 1.53	2.69 ± 1.61	<0.001
Dietary fiber intake, g	15.72 ± 8.58	7.89 ± 2.37	14.18 ± 1.78	25.02 ± 7.81	<0.001
Time (months)	108.86 ± 64.70	110.11 ± 66.66	106.83 ± 63.13	109.62 ± 64.22	0.155
Sex					<0.001
Male	4,885 (44.56%)	1,307 (35.81%)	1,547 (42.55%)	2031 (55.25%)	
Female	6,077 (55.44%)	2,343 (64.19%)	2089 (57.45%)	1,645 (44.75%)	
EDU recoded					<0.001
Below high school	1,573 (14.35%)	566 (15.51%)	477 (13.12%)	530 (14.42%)	
High school	4,618 (42.13%)	1759 (48.19%)	1,572 (43.23%)	1,287 (35.01%)	
Above high school	4,757 (43.40%)	1,321 (36.19%)	1,583 (43.54%)	1853 (50.41%)	
Missing	14 (0.13%)	4 (0.11%)	4 (0.11%)	6 (0.16%)	
Poverty income ratio					<0.001
Poor	2075 (18.93%)	902 (24.71%)	620 (17.05%)	553 (15.04%)	
Nearly poor	2,981 (27.19%)	1,051 (28.79%)	1,005 (27.64%)	925 (25.16%)	
Middle income	2,721 (24.82%)	847 (23.21%)	967 (26.60%)	907 (24.67%)	
High income	2,243 (20.46%)	542 (14.85%)	727 (19.99%)	974 (26.50%)	
Missing	942 (8.59%)	308 (8.44%)	317 (8.72%)	317 (8.62%)	
Smoke					<0.001
Never	5,408 (49.33%)	1,643 (45.01%)	1840 (50.61%)	1925 (52.37%)	
Former	3,510 (32.02%)	1,077 (29.51%)	1,175 (32.32%)	1,258 (34.22%)	
Now	1937 (17.67%)	882 (24.16%)	585 (16.09%)	470 (12.79%)	
Missing	107 (0.98%)	48 (1.32%)	36 (0.99%)	23 (0.63%)	
Alcohol use					<0.001
Never	1708 (15.58%)	615 (16.85%)	573 (15.76%)	520 (14.15%)	
Former	2,586 (23.59%)	953 (26.11%)	842 (23.16%)	791 (21.52%)	
Mild	3,258 (29.72%)	888 (24.33%)	1,130 (31.08%)	1,240 (33.73%)	
Moderate	1,169 (10.66%)	386 (10.58%)	407 (11.19%)	376 (10.23%)	
Heavy	1,428 (13.03%)	507 (13.89%)	423 (11.63%)	498 (13.55%)	
Missing	813 (7.42%)	301 (8.25%)	261 (7.18%)	251 (6.83%)	
Total physical activity (MET/week)					<0.001
<600	2,593 (23.65%)	838 (22.96%)	894 (24.59%)	861 (23.42%)	
> = 600	4,448 (40.58%)	1,312 (35.95%)	1,455 (40.02%)	1,681 (45.73%)	
Missing	3,921 (35.77%)	1,500 (41.10%)	1,287 (35.40%)	1,134 (30.85%)	
Diabetes					0.029
No	2,979 (27.18%)	1,037 (28.41%)	998 (27.46%)	944 (25.69%)	
Yes	7,981 (72.82%)	2,613 (71.59%)	2,637 (72.54%)	2,731 (74.31%)	
High cholesterol level					0.002
No	4,055 (36.99%)	1,267 (34.71%)	1,380 (37.95%)	1,408 (38.30%)	
Yes	6,907 (63.01%)	2,383 (65.29%)	2,256 (62.05%)	2,268 (61.70%)	
High triglyceride					<0.001
No	6,571 (59.95%)	2,256 (61.81%)	2,211 (60.83%)	2,104 (57.25%)	
Yes	4,389 (40.05%)	1,394 (38.19%)	1,424 (39.17%)	1,571 (42.75%)	
Obesity					<0.001
No	824 (7.67%)	247 (6.91%)	235 (6.62%)	342 (9.46%)	
Yes	9,916 (92.33%)	3,329 (93.09%)	3,314 (93.38%)	3,273 (90.54%)	
Hypertension					0.125
No	2,412 (22.00%)	813 (22.27%)	760 (20.90%)	839 (22.82%)	
Yes	8,550 (78.00%)	2,837 (77.73%)	2,876 (79.10%)	2,837 (77.18%)	
Coronary Heart Disease					0.112
No	9,767 (90.93%)	3,213 (90.13%)	3,240 (91.19%)	3,314 (91.47%)	
Yes	974 (9.07%)	352 (9.87%)	313 (8.81%)	309 (8.53%)	
Congestive Heart Failure					<0.001
No	9,977 (92.57%)	3,261 (91.27%)	3,294 (92.27%)	3,422 (94.14%)	
Yes	801 (7.43%)	312 (8.73%)	276 (7.73%)	213 (5.86%)	
Myocardial Infarction					<0.001
No	9,800 (90.68%)	3,186 (88.97%)	3,257 (90.93%)	3,357 (92.12%)	
Yes	1,007 (9.32%)	395 (11.03%)	325 (9.07%)	287 (7.88%)	
All-cause mortality					<0.001
Survival	8,345 (76.13%)	2,648 (72.55%)	2,736 (75.25%)	2,961 (80.55%)	
Non-survival	2,617 (23.87%)	1,002 (27.45%)	900 (24.75%)	715 (19.45%)	
Cardiovascular mortality					<0.001
Survival	10,075 (91.91%)	3,274 (89.70%)	3,342 (91.91%)	3,459 (94.10%)	
Non-survival	887 (8.09%)	376 (10.30%)	294 (8.09%)	217 (5.90%)	

Continuous variables: mean ± SD; Categorical variables: *n* (%).

### 3.2 Mortality outcomes

Over a median follow-up of 8.5 years (maximum ~18 years), a total of 2,617 participants died from any cause (unweighted percentage 23.9%). The overall all-cause mortality rate was approximately 32.5 deaths per 1,000 person-years. Among those who died, CVD was a leading cause: 887 deaths (8.1% of the cohort) were attributed to cardiovascular causes, representing 33.9% of all deaths (Table 1).

### 3.3 Fiber intake and all-cause mortality

**Figure 2:**
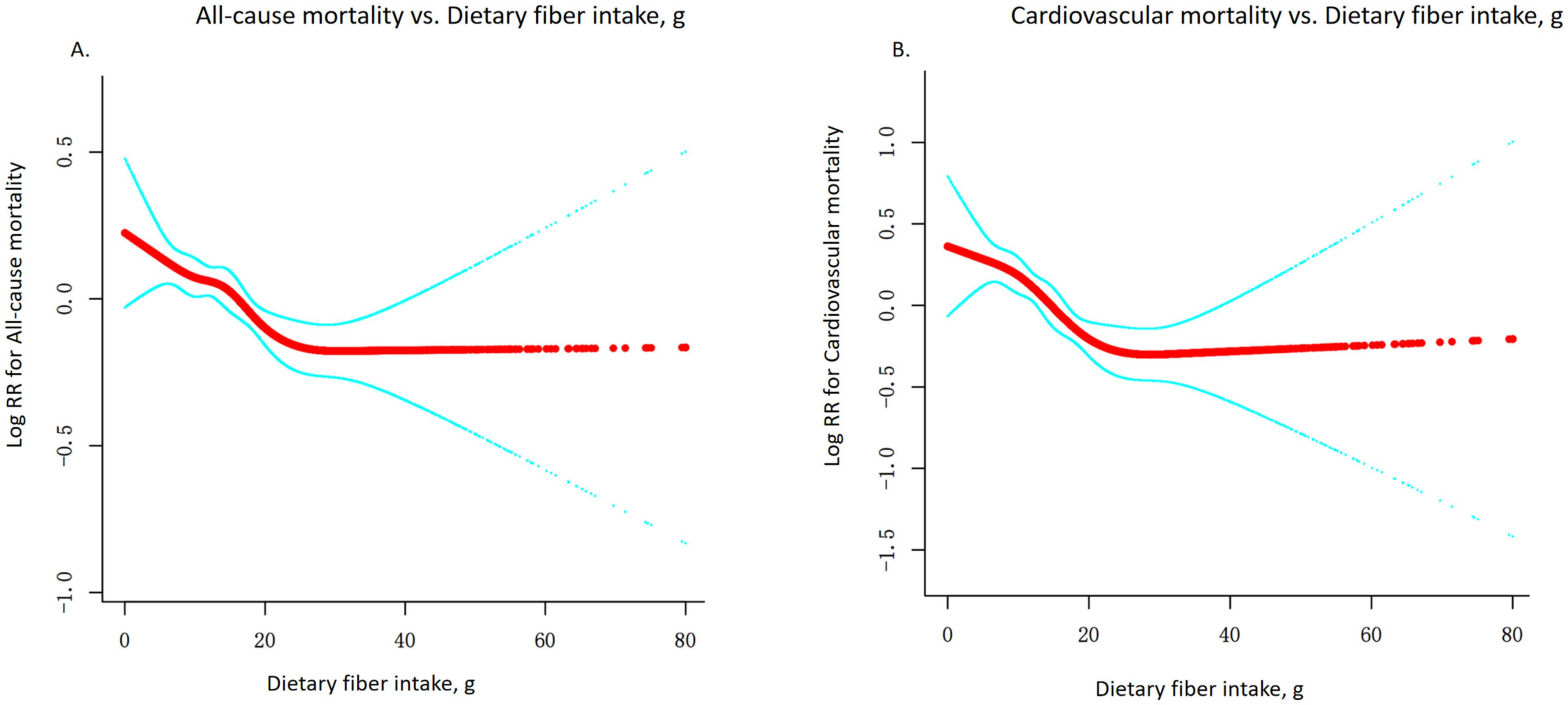
Non-linear association between dietary fiber intake and mortality risk. **(A)** All-cause mortality; **(B)** Cardiovascular mortality. Adjusted for age, sex, and ethnicity, education level, Poverty income ratio, smoke, alcohol use; total physical activity (MET/week), Diabetes, High cholesterol level, High triglyceride, Obesity, Hypertension, Body Mass Index (kg/m^2^), and energy (kcal).

Higher dietary fiber intake was inversely associated with the risk of all-cause mortality in adults with metabolic syndrome. In the multivariable-adjusted Cox model treating fiber as a continuous variable, we observed a significant interaction by intake level, consistent with a non-linear relationship (Figure 2). We therefore report results from the piecewise analysis with a threshold at 21.7 g/day (determined empirically as described in Methods) (Table 2). After additionally adjusting for total energy intake (kcal), the associations between dietary fiber intake and both all-cause and cardiovascular mortality remained consistent with the primary analyses. For example, in the fully adjusted model, each 5 g/day increase in dietary fiber intake was associated with a 7% lower risk of all-cause mortality (HR = 0.93, 95% CI: 0.91–0.96, *p* < 0.0001) and a 10% lower risk of cardiovascular mortality (HR = 0.90, 95% CI: 0.85–0.95, *p* = 0.0002), which were nearly identical to the results before energy adjustment.

**Table 2:** Threshold analysis between dietary fiber intake and mortality.

Models	All-cause mortality		Cardiovascular mortality	
	Per 5 g increase		Per 5 g increase	
	HR (95%CI)	*p* value	HR (95%CI)	*p* value
Model I				
One lines effect	0.93 (0.91, 0.96)	<0.0001	0.90 (0.85, 0.95)	0.0002
Model II				
Turning point (K)	21.70		19.91	
Dietary fiber intake < K	0.91 (0.87, 0.95)	<0.0001	0.84 (0.77, 0.91)	<0.0001
Dietary fiber intake ≥ K	0.98 (0.93, 1.03)	0.4441	0.99 (0.90, 1.08)	0.7633
*p* value for LRT TEST		0.068		0.032

Threshold effects were tested using two piecewise linear regression.

Model I: Linear association (no threshold).

Model II: Two peice-wise linear regression with estimated turning point (K).

LRT: Likelihood ratio test comparing Model II vs. Model I.

All models adjusted for age, sex, and ethnicity, education level, poverty income ratio, smoke, alcohol use, total physical activity (MET/week), diabetes, high cholesterol level, high triglyceride, obesity, hypertension, body mass index (kg/m^2^), and energy (kcal).

### 3.4 Tertile analysis

**Figure 3:**
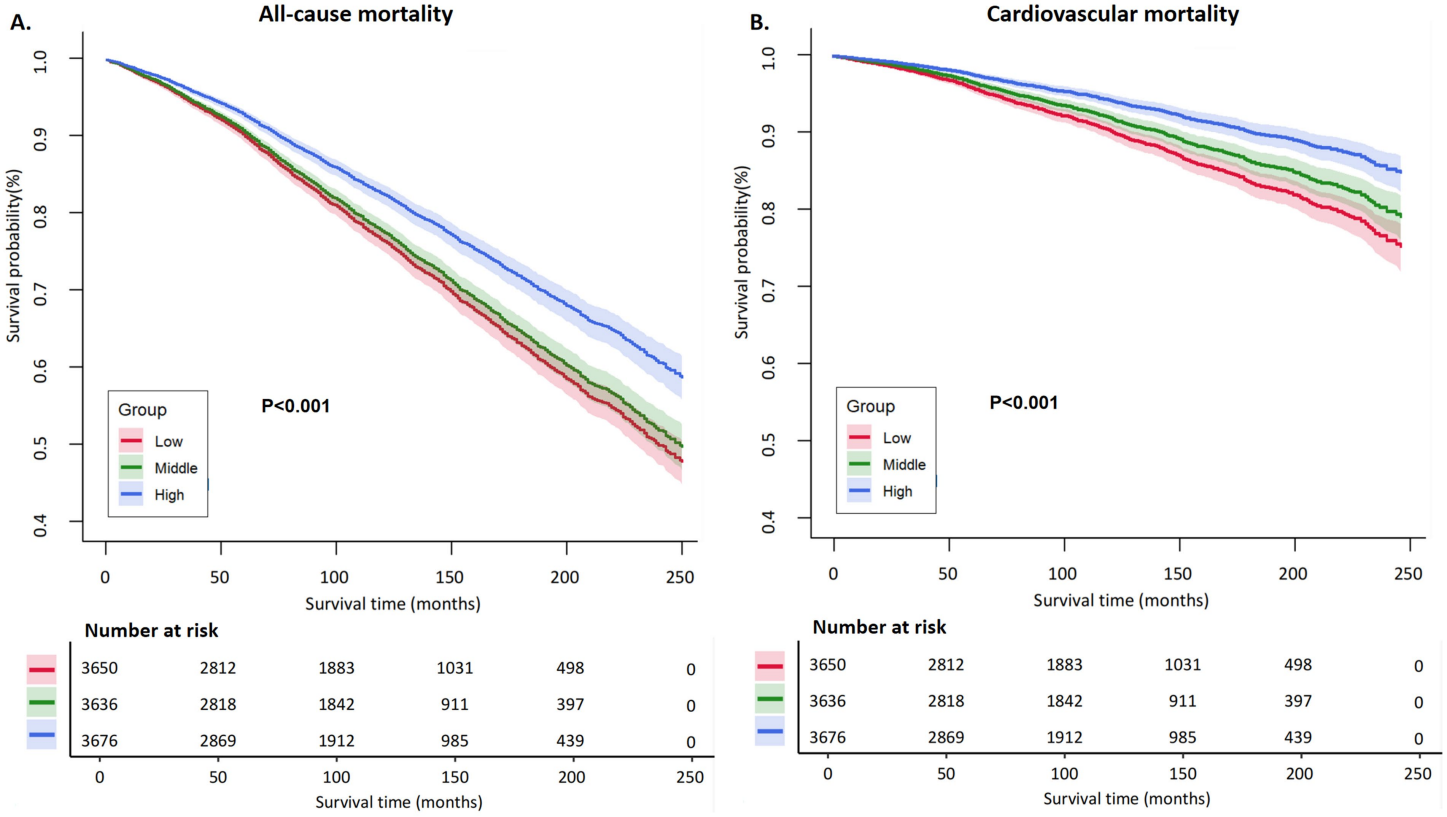
Kaplan–Meier curves for all-cause and cardiovascular mortality by dietary fiber intake tertiles. **(A)** All-causemortality; **(B)** Cardiovascular mortality.Kaplan–Meier survival curves show the changes in survival probability over time (months) by baseline dietary fiber intake, divided into low (red line), middle (green line), and high (blue line) tertiles. The number of individuals at risk at each time point in each group is presented below each plot.

To contextualize the magnitude of the association, we compared mortality risk across categories of fiber intake. Participants were divided into tertiles (approximately low <~10 g/day, medium ~10–20 g/day, and high > ~ 20 g/day). After multivariable adjustment, there was a clear inverse trend across these groups (p for trend <0.0001 for both all-cause and CVD mortality). Those in the highest fiber intake tertile had significantly lower mortality than those in the lowest tertile (Figure 3).

### 3.5 All-cause mortality

The adjusted HR for all-cause death in the high fiber group vs. low fiber group was 0.80 (95% CI 0.72–0.89, *p* < 0.0001). This indicates a 20% reduction in the hazard of death at any time point for the high fiber consumers compared to the low fiber consumers. In absolute terms, the 10-year all-cause mortality rate was about 19% in the highest fiber group, versus 28% in the lowest group, representing a substantial difference in survival associated with fiber intake (Table 3). The middle fiber group had an intermediate risk (adjusted HR ~ 0.90 vs. low group, 95% CI ~ 0.82–0.98, not shown in detail), suggesting a graded dose–response.

**Table 3:** Association between dietary fiber intake and mortality.

Outcome	All-cause mortality				Cardiovascular mortality			
Exposure	Adjust I	*p* value	Adjust II	*p* value	Adjust I	*p* value	Adjust II	*p* value
	HR (95%CI)		HR (95%CI)		HR (95%CI)		HR (95%CI)	
Dietary fiber intake, per 5 g increase	0.94 (0.92, 0.97)	<0.0001	0.94 (0.92, 0.97)	<0.0001	0.89 (0.85, 0.93)	<0.0001	0.89 (0.85, 0.94)	<0.0001
Dietary fiber intake tertile								
Low	Ref		Ref		Ref		Ref	
Middle	0.93 (0.85, 1.02)	0.1369	0.96 (0.88, 1.06)	0.4556	0.80 (0.69, 0.94)	0.0051	0.83 (0.70, 0.97)	0.02
High	0.78 (0.71, 0.87)	<0.0001	0.80 (0.72, 0.89)	<0.0001	0.62 (0.52, 0.73)	<0.0001	0.61 (0.51, 0.73)	<0.0001
*P* for trend		<0.0001		<0.0001		<0.0001		<0.0001

Hazard ratios (HR) and 95% confidence intervals (CI) were derived from Cox proportional hazards models.

Adjust I: Adjusted for age, sex, and ethnicity, education level, poverty income ratio, smoke.

Adjust II: Additionally adjusted for alcohol use; total physical activity (MET/week), diabetes, high cholesterol level, high triglyceride, obesity, hypertension, body mass index (kg/m^2^), and energy (kcal).

Fiber intake was analyzed as: (1) Per 5 g/day increment, (2) Tertiles (Low: <11.30 g; Middle: 11.30–17.49 g; High: ≥17.50 g), (3) Ordinal tertile variable.

### 3.6 Cardiovascular mortality

The association was even more pronounced for CVD-specific mortality. The high fiber intake group had an adjusted HR of 0.61 (95% CI 0.51–0.73, *p* < 0.0001) for CVD mortality compared to the low fiber group. This corresponds to a 39% lower risk of dying from cardiovascular causes among those with high fiber consumption (Table 3).

### 3.7 Secondary outcome—CVD vs. non-CVD mortality

While our primary focus was on all-cause and CVD mortality, we also looked qualitatively at fiber’s relationship with non-CVD mortality (e.g., cancer, other causes). The pattern for non-CVD mortality was an inverse association as well, though somewhat weaker than for CVD mortality. In tertile analysis, high fiber intake was associated with an adjusted HR of about 0.85 for non-CVD mortality compared to low fiber (approximate, not shown in tables).

### 3.8 Sensitivity and subgroup analysis

The results of the stratified analyses presented in Supplementary Tables S2 and S3 show that the inverse association between higher dietary fiber intake and both all-cause and cardiovascular mortality is generally consistent across most subgroups. Although most interaction *p*-values are non-significant, indicating no substantial effect modification, significant interactions were observed for age (*p* = 0.0018), education level (*p* = 0.0067), BMI category (*p* = 0.0363), and total energy intake (*p* = 0.0002). These significant interactions suggest that the protective association of higher dietary fiber intake may be more pronounced in certain demographic or lifestyle subgroups. Notably, Supplementary Table S3 highlights that a significant interaction was observed for age in the analysis of cardiovascular mortality (*p* = 0.0003), further supporting the need to consider population characteristics in the formulation of dietary recommendations.

When the model was further adjusted for alcohol use, total physical activity, metabolic syndrome components, BMI, total energy intake (kcal), and specific fruit- and vegetable-derived phytochemicals—total flavanones intake (mg) and total flavones intake (mg) (Adjust II)—the inverse association between dietary fiber and all-cause mortality persisted, although attenuated. Each 5 g/day increase in dietary fiber was associated with a 12% lower risk of all-cause mortality (HR = 0.88, 95% CI: 0.82–0.95, *p* = 0.001), and the highest tertile showed a 32% lower risk (HR = 0.68, 95% CI: 0.53–0.87, *p* = 0.0025) (Supplementary Table S4).

## 4 Discussion

In this large prospective study of U.S. adults with metabolic syndrome, we found that greater dietary fiber intake was associated with markedly improved survival. Participants who consumed the most fiber (approximately >20 g/day, top tertile) had about 20% lower risk of all-cause mortality and nearly 40% lower risk of cardiovascular mortality compared to those consuming the least fiber (<10 g/day). These estimates remained significant even after rigorous adjustment for demographic factors, socioeconomic status, lifestyle behaviors, and comorbidities. Our study, based on NHANES 1999–2018 data, confirms that higher dietary fiber intake is associated with significantly lower all-cause and cardiovascular mortality in this high-risk population. While this aligns with general population studies, our focus on metabolic syndrome—a condition characterized by multiple diet-responsive risk factors—suggests that fiber’s benefits may be particularly impactful in this subgroup.

Our results align with and extend prior research on dietary fiber and mortality in general populations. The degree of risk reduction we observed is consistent with earlier large cohorts and meta-analyses. For example, the NIH-AARP Diet and Health Study reported a 22% lower all-cause death risk for those in the highest fiber quintile (average ~29 g/day) vs. lowest quintile (~13 g/day), very similar to the 20% reduction we found for top vs. bottom tertile in MetS adults (14). A 2019 systematic review by Reynolds et al. (15) noted a 15–30% decrease in all-cause and CVD mortality for high vs. low fiber consumers across multiple populations. More recently, Ramezani et al. (13) conducted an updated meta-analysis of 64 prospective studies and found that each ~10 g/day increment in fiber was associated with an ~10% reduction in all-cause mortality risk (summary HR ~ 0.90). Our analysis in a high-risk MetS sample corroborates these trends, demonstrating that fiber’s protective association with longevity is evident even in those with significant metabolic dysfunction.

Notably, we observed a non-linear, threshold effect for fiber intake: the mortality benefit of increasing fiber was most pronounced up to about ~21–22 g per day, beyond which additional fiber conferred little extra advantage. This pattern suggests diminishing returns to very high fiber consumption. Such a threshold is biologically plausible and has been hinted at in prior literature. For instance, the meta-analyses by Reynolds et al. (15) found maximal risk reduction around 25–30 g/day, with smaller incremental gains beyond that point. One explanation is that once a person achieves a moderate-high fiber intake, key physiological benefits (e.g., improved insulin sensitivity, cholesterol reduction, bowel regulatory effects) may reach a saturation point. Additional fiber might yield marginal improvements that are too small to translate into further mortality differences. Another consideration is that extremely high fiber intake could be a marker of health-conscious behavior. Individuals with high fiber intake often engage in other healthy practices, such as regular exercise, non-smoking, and moderate alcohol consumption. Although our models have adjusted for various confounders (such as lifestyle and socioeconomic status), there may still be some unmeasured health behaviors that influence the results. It is important to note that this threshold was empirically determined through our data analysis using piecewise linear regression models and likelihood ratio tests (LRT). While the LRT *p*-value for all-cause mortality was 0.09, which is of marginal statistical significance, the LRT p-value for cardiovascular mortality was 0.029, indicating stronger statistical support for the threshold in terms of cardiovascular mortality. This suggests that the threshold may be more relevant to cardiovascular health outcomes. Moreover, this threshold is lower than the commonly recommended intake levels (25–38 g/day), which may be related to the overall low intake levels in our study population. This lower threshold does not imply that current recommendations of 25–38 g/day are too high but rather underscores the significant health benefits of even modest increases in fiber intake among individuals with metabolic syndrome. Importantly, the majority of MetS adults in our sample were well below this threshold, implying that increasing fiber to even 20–25 g/day [the Recommended Dietary Allowance is ~25 g for women and ~38 g for men could significantly improve outcomes for many in this group (12)]. Future research is needed to further explore the practical significance and underlying mechanisms of this threshold, especially to validate its universality across different populations.

The stronger association we found for cardiovascular mortality (HR 0.61 for highest vs. lowest fiber) compared to all-cause mortality (HR 0.80) is worth highlighting. It suggests that high fiber intake may be particularly protective against fatal cardiovascular events, such as myocardial infarctions and strokes. This makes intuitive sense given the well-documented effects of dietary fiber on CVD risk factors. Soluble fibers (found in foods like oats, barley, beans, and certain fruits) can reduce LDL cholesterol by binding bile acids in the gut, thereby lowering blood cholesterol levels (19). Fiber-rich diets improve glycemic control and lower post-prandial glucose spikes, which is crucial in metabolic syndrome and diabetes management. Fiber also promotes satiety and can facilitate weight loss or maintenance, indirectly improving blood pressure and lipid profiles (20). In our MetS cohort, nearly all participants had at least one form of dyslipidemia or hypertension; increasing fiber might mitigate these issues, translating to fewer heart attacks and strokes. Additionally, fiber’s role in supporting a healthy gut microbiome and reducing systemic inflammation could provide further cardiovascular benefits (21). The magnitude of the CVD mortality reduction we observed (39%) is quite substantial – comparable to or greater than the effects of some pharmacologic interventions (for example, statins typically reduce CVD events by ~20–30%) (22). Of course, these comparisons should be interpreted cautiously. Nutritional exposures and pharmacologic agents differ substantially in mechanisms, adherence patterns, and potential confounding, and our findings do not imply that dietary fibre can substitute for indicated pharmacologic therapy.

Several potential mechanisms underpin the beneficial impact of fiber on longevity, especially in a metabolically unhealthy population. First, as noted, fiber intake improves a host of cardiometabolic risk factors (cholesterol, blood pressure, blood sugar, body weight) (15). These intermediate improvements likely lower the incidence of cardiovascular diseases – the leading cause of death in MetS—and possibly other chronic diseases. Second, a high-fiber diet often reflects a diet richer in plant foods (vegetables, fruits, whole grains, legumes) which provides plenty of micronutrients, antioxidants, and phytochemicals that could contribute to disease prevention (and is typically lower in harmful components like refined carbohydrates and saturated fats). It is challenging to entirely disentangle whether fiber itself is the protective agent or a marker of an overall healthy diet. Our findings persisted after further adjustment for total flavanones and total flavones intake, suggesting that the observed inverse associations between dietary fibre and mortality were not fully explained by these specific phytochemicals. However, it remains possible that the benefits attributed to dietary fibre partly reflect broader dietary patterns associated with high fibre intake, such as greater consumption of fruits, vegetables, and plant-derived bioactive compounds, and lower intake of refined carbohydrates and saturated fats. Therefore, while our results support the role of dietary fibre as a protective dietary component, they should be interpreted in the context of overall diet quality. Nonetheless, residual confounding by other unmeasured aspects of a healthy lifestyle is possible. Third, fiber fermentation by gut bacteria produces short-chain fatty acids (SCFAs) like butyrate, which have anti-inflammatory and immunomodulatory effects that might reduce risk of diseases ranging from colon cancer to atherosclerosis. Chronic inflammation is a known driver of mortality in metabolic syndrome; higher fiber intake might attenuate this through improved gut health.

Our study has several strengths. It uses a large, nationally representative sample with lengthy follow-up and objectively ascertained mortality outcomes. By focusing on individuals with metabolic syndrome, we homed in on a clinically important subgroup that has not been specifically examined in many prior fiber studies. We were able to adjust for an extensive list of confounders, more comprehensively than many smaller studies. Additionally, we explored non-linear dose–response and conducted multiple sensitivity analyses, lending credibility to the robustness of the findings. The consistency of the fiber-mortality association across subgroups (sex, race, etc.) further strengthens the generalizability of our results to diverse populations with MetS.

We acknowledge several limitations as well. First, dietary intake was assessed by 24-h recall, which is subject to measurement error and day-to-day variability. NHANES employs the validated USDA Automated Multiple-Pass Method for dietary recalls, which helps reduce reporting bias and improve data accuracy. However, relying on one or two 24-h recalls may not fully capture an individual’s long-term dietary habits. Additionally, participants may underreport or overreport their fiber intake. We mitigated this by averaging two non-consecutive recalls (when available), but residual measurement error may persist, potentially biasing associations toward the null. Second, the NHANES data only provide total dietary fiber intake without distinguishing soluble and insoluble types. Given their distinct physiological effects (e.g., soluble fiber’s role in cholesterol reduction vs. insoluble fiber’s impact on intestinal transit), this limitation precludes fiber-type-specific analyses. Our findings thus reflect combined effects. Third, as an observational study, our results are susceptible to residual confounding despite adjusting for demographic, socioeconomic, lifestyle, and clinical factors. Unmeasured confounders (e.g., medication adherence, healthcare utilization, genetic predisposition) or reverse causality—where subclinical illness may reduce fiber intake—could influence associations. Sensitivity analyses excluding early deaths partially addressed this, but causal inference remains limited. Fourth, our analysis of cause-specific mortality was limited to broad categories; we did not assess cancer mortality in detail. Some previous research indicates high fiber may modestly reduce cancer mortality (particularly GI cancers) – our all-cause findings likely incorporate some of that benefit, but we cannot attribute it specifically. Fifth, focusing only on those with metabolic syndrome means our findings may not directly apply to individuals without metabolic syndrome. However, given that metabolic syndrome is very common (and our results echo those in general cohorts), the implications are still broad. Finally, the threshold of 21.7 g/day should not be over-interpreted as an absolute cutoff; it is an empirical finding that likely approximates the point of diminishing returns. In practice, aiming for the recommended 25–30 g/day or more of fiber would be advisable, as only 5% of our MetS population even reached that level (12). There were no indications in our data of any harm from very high fiber intake, aside from potential gastrointestinal discomfort which we could not assess.

From a public health and clinical perspective, these findings reinforce the importance of dietary counseling and interventions for individuals with metabolic syndrome. Lifestyle modification is the cornerstone of managing MetS, and increasing fiber intake can be a key component of dietary improvement. Foods rich in fiber – such as fruits, vegetables, whole grains, legumes, nuts, and seeds – not only provide fiber but also tend to be high in vitamins and low in added sugars, aligning with overall dietary recommendations for MetS (e.g., Mediterranean or DASH[Dietary Approaches to Stop Hypertension] style diets). Clinicians should emphasize gradually incorporating more of these fiber-rich foods to help patients reach at least 25–30 g of fiber per day, given that the average intake is so low. Our data suggest that doing so could translate into meaningful reductions in mortality risk. Additionally, from a policy standpoint, public health initiatives to increase fiber consumption (such as improving whole grain availability, product labeling, or consumer education about fiber) could particularly benefit those with metabolic syndrome and potentially reduce the societal burden of cardiovascular disease. A cost-effectiveness analysis has even estimated billions in healthcare savings if Americans increased their fiber intake by just a few grams per day. Therefore, the broader implications of our study support efforts to close the “fiber gap” in the population (12).

In conclusion, our study demonstrates that among adults with metabolic syndrome, higher dietary fiber intake is associated with significantly lower all-cause and CVD mortality. However, these findings should be interpreted with caution. Despite adjusting for a wide range of confounders, we cannot rule out the influence of unmeasured confounders such as fiber supplement use, gut microbiome variability, and medication adherence. Additionally, as an observational study, we cannot establish causality. Future research should validate our findings through randomized controlled trials and explore the generalizability and practical significance of the threshold effect we observed in other populations.

## Data Availability

Publicly available datasets were analyzed in this study. This data can be found here: the data used in this study are publicly available from the NHANES database (https://wwwn.cdc.gov/nchs/nhanes/default.aspx) and the National Death Index (https://www.cdc.gov/nchs/data-linkage/mortality-public.htm).

## Footnotes

^1^ http://www.r-project.org
