# Supplementary material for: Association of dietary fiber intake with all-cause and cardiovascular mortality in U.S. adults with metabolic syndrome: NHANES 1999–2018
Source: Front Nutr. 2025 Sep 11;12:1659000. doi: 10.3389/fnut.2025.1659000 (PMC12460082; doi:10.3389/fnut.2025.1659000)
Supplement: Supplementary file 1 [file Table_1.DOCX]

Supplementary Material

# Supplementary Figures and Tables

## Supplementary Tables

**Table S1.** Detailed Subgroup Characteristics by Dietary Fiber Intake Tertiles

| Characteristics | Total | Dietary fiber intake, g/day | | | *P*-value |
| --- | --- | --- | --- | --- | --- |
|  |  | Tertile1 0.00-11.29 | Tertile 2 11.30-17.49 | Tertile 3 17.50-80.00 |  |
| Number | 10962 | 3650 | 3636 | 3676 |  |
| Ethnicity |  |  |  |  | <0.001 |
| Non-Hispanic White | 5232 (47.73%) | 1689 (46.27%) | 1837 (50.52%) | 1706 (46.41%) |  |
| Non-Hispanic Black | 2225 (20.30%) | 1001 (27.42%) | 717 (19.72%) | 507 (13.79%) |  |
| Mexican American | 1988 (18.14%) | 493 (13.51%) | 567 (15.59%) | 928 (25.24%) |  |
| Other Hispanic | 876 (7.99%) | 296 (8.11%) | 304 (8.36%) | 276 (7.51%) |  |
| Other Race | 641 (5.85%) | 171 (4.68%) | 211 (5.80%) | 259 (7.05%) |  |
| Marital status |  |  |  |  | <0.001 |
| Married/Living with Partner | 6559 (60.73%) | 2023 (56.51%) | 2117 (59.12%) | 2419 (66.46%) |  |
| Widowed/Divorced/Separated | 3164 (29.29%) | 1141 (31.87%) | 1127 (31.47%) | 896 (24.62%) |  |
| Never married | 1078 (9.98%) | 416 (11.62%) | 337 (9.41%) | 325 (8.93%) |  |

Continuous variables: mean ± SD ; Categorical variables: n (%) .

**Table S2.** Stratified analyses of the association between high versus low dietary fiber intake and all-cause mortality in U.S. adults with metabolic syndrome

| Covariates | HR (95% CI) | *P*-value | *P* for interaction |
| --- | --- | --- | --- |
| Age (years) |  |  | 0.0018 |
| Low | 0.71 (0.54, 0.93) | 0.0142 |  |
| Middle | 0.62 (0.53, 0.73) | <0.0001 |  |
| High | 0.86 (0.78, 0.94) | 0.0015 |  |
| Sex |  |  | 0.2226 |
| Male | 0.74 (0.67, 0.83) | <0.0001 |  |
| Female | 0.82 (0.73, 0.92) | 0.0005 |  |
| Ethnicity |  |  | 0.0940 |
| Non-Hispanic White | 0.84 (0.76, 0.93) | 0.0005 |  |
| Non-Hispanic Black | 0.92 (0.76, 1.11) | 0.3681 |  |
| Mexican American | 0.64 (0.52, 0.79) | <0.0001 |  |
| Other Hispanic | 0.95 (0.66, 1.38) | 0.8077 |  |
| Other Race | 0.91 (0.56, 1.47) | 0.6919 |  |
| EDU recoded |  |  | 0.0067 |
| Below high school | 0.66 (0.56, 0.78) | <0.0001 |  |
| High school | 0.92 (0.82, 1.03) | 0.1577 |  |
| Above high school | 0.86 (0.76, 0.99) | 0.034 |  |
| Poverty income ratio |  |  | 0.5383 |
| Poor | 0.82 (0.69, 0.99) | 0.0344 |  |
| Nearly poor | 0.88 (0.77, 1.00) | 0.054 |  |
| Middle income | 0.92 (0.79, 1.07) | 0.2871 |  |
| High income | 0.85 (0.69, 1.04) | 0.1207 |  |
| Missing | 0.70 (0.54, 0.92) | 0.0105 |  |
| Smoke |  |  | 0.3691 |
| Never | 0.79 (0.70, 0.89) | 0.0001 |  |
| Former | 0.79 (0.70, 0.89) | <0.0001 |  |
| Now | 0.89 (0.74, 1.09) | 0.2557 |  |
| Alcohol use |  |  | 0.3299 |
| Never | 0.75 (0.62, 0.90) | 0.0021 |  |
| Former | 0.89 (0.78, 1.01) | 0.0724 |  |
| Mild | 0.86 (0.74, 1.00) | 0.0434 |  |
| Moderate | 0.68 (0.50, 0.93) | 0.015 |  |
| Heavy | 0.72 (0.54, 0.95) | 0.0204 |  |
| Missing | 0.88 (0.59, 1.32) | 0.538 |  |
| Total physical activity (MET/week) |  |  | 0.2175 |
| <600 | 0.95 (0.82, 1.10) | 0.5025 |  |
| >=600 | 0.80 (0.69, 0.93) | 0.0046 |  |
| Missing | 0.83 (0.75, 0.93) | 0.0014 |  |
| Diabetes |  |  | 0.2725 |
| No | 0.74 (0.63, 0.88) | 0.0004 |  |
| Yes | 0.83 (0.76, 0.90) | <0.0001 |  |
| High cholesterol level |  |  | 0.3143 |
| No | 0.85 (0.76, 0.96) | 0.0068 |  |
| Yes | 0.78 (0.71, 0.87) | <0.0001 |  |
| High triglyceride |  |  | 0.1650 |
| No | 0.86 (0.78, 0.95) | 0.0031 |  |
| Yes | 0.77 (0.68, 0.87) | <0.0001 |  |
| Obesity |  |  | 0.0655 |
| No | 0.64 (0.50, 0.83) | 0.0008 |  |
| Yes | 0.83 (0.76, 0.90) | <0.0001 |  |
| Hypertension |  |  | 0.4618 |
| No | 0.76 (0.60, 0.96) | 0.0199 |  |
| Yes | 0.82 (0.76, 0.89) | <0.0001 |  |
| Body Mass Index(kg/m2) |  |  | 0.0363 |
| Low | 0.79 (0.70, 0.89) | <0.0001 |  |
| Middle | 0.72 (0.63, 0.83) | <0.0001 |  |
| High | 0.95 (0.82, 1.11) | 0.5212 |  |
| Energy |  |  | 0.0002 |
| Low | 0.92 (0.80, 1.06) | 0.2718 |  |
| Middle | 1.24 (1.08, 1.41) | 0.0016 |  |
| High | 0.81 (0.68, 0.96) | 0.0127 |  |

Hazard ratios (HRs) compare high versus low dietary fiber intake within each subgroup.

**Table S3.** Stratified analyses of the association between high versus low dietary fiber intake and cardiovascular mortality in U.S. adults with metabolic syndrome

| Covariates | HR (95% CI) P-value | *P* for interaction |
| --- | --- | --- |
| Age (years) |  | 0.0003 |
| Low | 0.50 (0.29, 0.84) 0.0095 |  |
| Middle | 0.42 (0.30, 0.58) <0.0001 |  |
| High | 0.82 (0.70, 0.96) 0.0119 |  |
| Sex |  | 0.9900 |
| Male | 0.68 (0.57, 0.82) <0.0001 |  |
| Female | 0.68 (0.56, 0.84) 0.0002 |  |
| Ethnicity |  | 0.7258 |
| Non-Hispanic White | 0.79 (0.67, 0.93) 0.0054 |  |
| Non-Hispanic Black | 0.69 (0.49, 0.96) 0.0289 |  |
| Mexican American | 0.70 (0.49, 1.00) 0.0475 |  |
| Other Hispanic | 0.54 (0.25, 1.14) 0.1049 |  |
| Other Race | 0.54 (0.19, 1.49) 0.2339 |  |
| EDU recoded |  | 0.1583 |
| Below high school | 0.59 (0.44, 0.79) 0.0004 |  |
| High school | 0.86 (0.70, 1.05) 0.1294 |  |
| Above high school | 0.72 (0.57, 0.91) 0.0057 |  |
| Poverty income ratio |  | 0.3183 |
| Poor | 0.67 (0.49, 0.92) 0.0141 |  |
| Nearly poor | 0.85 (0.68, 1.07) 0.1631 |  |
| Middle income | 0.74 (0.57, 0.97) 0.0262 |  |
| High income | 0.93 (0.65, 1.35) 0.7109 |  |
| Missing | 0.51 (0.30, 0.88) 0.0159 |  |
| Smoke |  | 0.3167 |
| Never | 0.64 (0.52, 0.78) <0.0001 |  |
| Former | 0.81 (0.66, 1.00) 0.0449 |  |
| Now | 0.59 (0.40, 0.88) 0.0090 |  |
| Alcohol use |  | 0.3521 |
| Never | 0.70 (0.51, 0.95) 0.0226 |  |
| Former | 0.83 (0.67, 1.03) 0.0916 |  |
| Mild | 0.76 (0.59, 0.97) 0.0285 |  |
| Moderate | 0.45 (0.25, 0.80) 0.0065 |  |
| Heavy | 0.58 (0.33, 1.03) 0.0630 |  |
| Missing | 0.62 (0.28, 1.39) 0.2471 |  |
| Total physical activity(MET/week) |  | 0.4512 |
| <600 | 0.73 (0.56, 0.96) 0.0226 |  |
| >=600 | 0.90 (0.69, 1.18) 0.4650 |  |
| Missing | 0.73 (0.60, 0.88) 0.0009 |  |
| Diabetes |  | 0.4838 |
| No | 0.66 (0.50, 0.87) 0.0037 |  |
| Yes | 0.74 (0.64, 0.86) <0.0001 |  |
| High cholesterol level |  | 0.8024 |
| No | 0.74 (0.60, 0.91) 0.0043 |  |
| Yes | 0.72 (0.60, 0.85) 0.0002 |  |
| High triglyceride |  | 0.4002 |
| No | 0.77 (0.65, 0.91) 0.0027 |  |
| Yes | 0.68 (0.55, 0.85) 0.0005 |  |
| Obesity |  | 0.3588 |
| No | 0.59 (0.38, 0.92) 0.0191 |  |
| Yes | 0.72 (0.63, 0.84) <0.0001 |  |
| Hypertension |  | 0.4658 |
| No | 0.90 (0.55, 1.46) 0.6573 |  |
| Yes | 0.72 (0.62, 0.82) <0.0001 |  |
| Body Mass Index(kg/m2) |  | 0.6175 |
| Low | 0.69 (0.55, 0.85) 0.0005 |  |
| Middle | 0.67 (0.53, 0.85) 0.0010 |  |
| High | 0.79 (0.61, 1.04) 0.0905 |  |
| Energy |  | 0.0701 |
| Low | 0.85 (0.67, 1.08) 0.1718 |  |
| Middle | 1.18 (0.94, 1.49) 0.1565 |  |
| High | 0.81 (0.59, 1.10) 0.1787 |  |

Hazard ratios (HRs) compare high versus low dietary fiber intake within each subgroup.

**Table S4.** Multivariable-adjusted hazard ratios (HRs) and 95% confidence intervals (CIs) for the association between dietary fiber intake and all-cause and cardiovascular mortality in U.S. adults with metabolic syndrome

| Outcome | Exposure | Adjust I HR (95% CI) | p-value | Adjust II HR (95% CI) | p-value |
| --- | --- | --- | --- | --- | --- |
| All-cause mortality | Dietary fiber intake (per 5 g/day) | 0.94 (0.92, 0.97)*** | <0.0001 | 0.88 (0.82, 0.95)** | 0.0010 |
|  | Low | 1.00 (ref) | – | 1.00 (ref) | – |
|  | Middle | 0.93 (0.85, 1.02) | 0.1369 | 0.95 (0.78, 1.16) | 0.6197 |
|  | High | 0.78 (0.71, 0.87)*** | <0.0001 | 0.68 (0.53, 0.87)** | 0.0025 |
|  | Tertile continuous | 0.89 (0.84, 0.93)*** | <0.0001 | 0.84 (0.74, 0.95)** | 0.0040 |
| Cardiovascular mortality | Dietary fiber intake (per 5 g/day) | 0.89 (0.85, 0.93)*** | <0.0001 | 0.90 (0.78, 1.04) | 0.1425 |
|  | Low | 1.00 (ref) | – | 1.00 (ref) | – |
|  | Middle | 0.80 (0.69, 0.94)** | 0.0051 | 1.00 (0.71, 1.41) | 0.9914 |
|  | High | 0.62 (0.52, 0.73)*** | <0.0001 | 0.59 (0.37, 0.94)* | 0.0249 |
|  | Tertile continuous | 0.79 (0.72, 0.86)*** | <0.0001 | 0.80 (0.64, 0.99)* | 0.0438 |

Adjust I model adjusted for age (years), sex, ethnicity, education level, poverty income ratio, and smoking.

Adjust II model adjusted for age (years), sex, ethnicity, education level ), poverty income ratio, smoking, alcohol use, total physical activity (MET-min/week), metabolic syndrome components, body mass index (kg/m²), total energy intake (kcal), total flavanones intake (mg), and total flavones intake (mg).
Cox proportional hazards models used follow-up time as the time scale.
Sample sizes: Adjust I: n = 10,962; Adjust II: n = 3,710 for each model.
p < 0.05*, p < 0.01**, p < 0.001***.

**
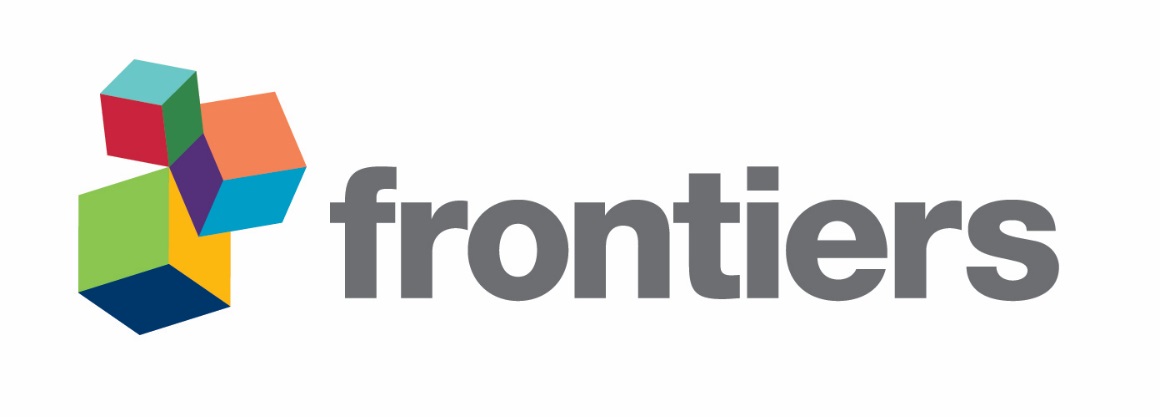
**
